# ^211^At-labeled immunoconjugate *via* a one-pot three-component double click strategy: practical access to α-emission cancer radiotherapeutics†
†Electronic supplementary information (ESI) available. See DOI: 10.1039/c8sc04747b


**DOI:** 10.1039/c8sc04747b

**Published:** 2018-12-21

**Authors:** Katsumasa Fujiki, Yousuke Kanayama, Shinya Yano, Nozomi Sato, Takuya Yokokita, Peni Ahmadi, Yasuyoshi Watanabe, Hiromitsu Haba, Katsunori Tanaka

**Affiliations:** a Biofunctional Synthetic Chemistry Laboratory , RIKEN Cluster for Pioneering Research , 2-1 Hirosawa , Wako , Saitama 351-0198 , Japan . Email: kotzenori@riken.jp; b GlycoTargeting Research Laboratory , RIKEN Baton Zone Program , 2-1 Hirosawa , Wako , Saitama 351-0198 , Japan; c Laboratory for Pathophysiological and Health Science , RIKEN Center for Biosystems Dynamics Research , 6-7-3 Minatojima-minamimachi, Chuo-ku , Kobe , Hyogo 650-0047 , Japan; d Nuclear Chemistry Research Team , RIKEN Nishina Center for Accelerator-Based Science , 2-1 Hirosawa , Wako , Saitama 351-0198 , Japan; e Biofunctional Chemistry Laboratory , A. Butlerov Institute of Chemistry , Kazan Federal University , 18 Kremlyovskaya Street , Kazan 420008 , Russia

## Abstract

α-Emission radiotherapeutics has potential to be one of most effective cancer therapeutics. Herein, we report a facile synthesis of an ^211^At-labeled immunoconjugate for use as an α-emission molecular targeting therapy. We synthesized a tetrazine probe modified with *closo*-decaborate(2-), a prosthetic group that forms a bioavailable stable complex with ^211^At. Our one-pot three-component double-click labeling method was used to attach decaborate to trastuzumab (anti-HER2 antibody) using decaborate-tetrazine and TCO-aldehyde probes without reducing the antibody binding affinity. Labeling the decaborate-attached trastuzumab with ^211^At produced in the cyclotron at the RIKEN Nishina Center, at which highly radioactive ^211^At can be produced, readily furnished the ^211^At-labeled trastuzumab with a maximum specific activity of 15 MBq μg^–1^ and retention of the native binding affinity. Intratumor injection of the ^211^At-labeled trastuzumab in BALB/c nude mice implanted with HER2-expressing epidermoid cancer cells yielded efficient accumulation at the targeted tumor site as well as effective suppression of tumor growth.

## Abstract 2

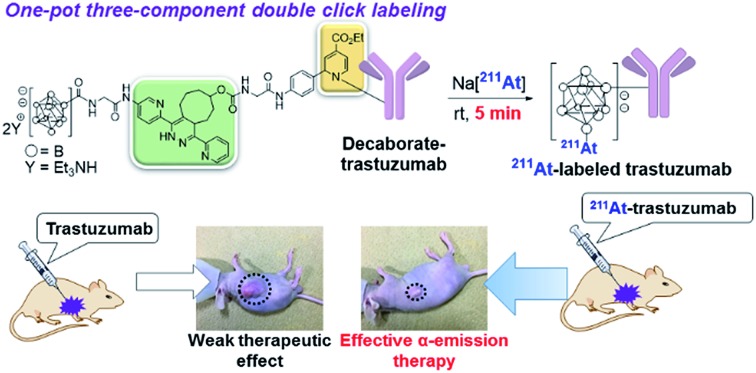
A facile synthesis of an ^211^At-labeled immunoconjugate for α-emission therapy was achieved based on a one-pot three-component double click method.

## Introduction

Radiation therapeutics, including radionuclides, show promise as effective cancer therapeutics. Radiolabeled cancer-targeting molecules have recently attracted attention in the field of radiopharmaceutical development.^1^–^4^ Several radioimmunoconjugates^5^–^7^ have been developed, including ^90^Y-labeled rituximab (an anti-CD20 antibody commercialized under the name Zevalin),^8^^131^I-labeled tositumomab (an anti-CD20 antibody commercialized under the name Bexxar),^9^ and ^177^Lu-labeled anti-CD20 and anti-CD37 antibodies^10^ for use in β-emitting therapy.

α-Emission radiotherapy has attracted attention for its potent cancer therapeutic efficacy. Radionuclides that decay by emitting alpha particles can damage targeted cancer tissue within a few cell diameters, thereby minimizing off-target damage. α-Emitters, including ^225^Ac, with a half-life of 10 days, ^223^Ra, with a half-life of 11.4 days, and ^227^Th, with a half-life of 18.7 days, are favored for use in radiotherapy techniques involving targeted molecules, such as radioimmunoconjugates, because of their long half-lives.^11^ Efficient chelators of these radionuclides must be developed before such applications may be realized.^12^,^13^ ^211^At, with a half-life of 7.2 h, is one of the most attractive α-particle nuclides for use in molecularly targeted therapy,^14^,^15^ and several approaches to preparing ^211^At-labeled molecules for cancer targeting have been explored. Astatination may be achieved using prosthetic groups such as stanylphenyl,^16^–^18^ silylphenyl,^19^ or borylphenyl^20^ moieties. Aryliodonium salt derivatives^21^ or caged carborane derivatives^22^–^25^ tend to be good chelators of ^211^At as well. The use of these labeling groups has enabled the manufacture of ^211^At-labeled molecular complexes that are stable to metabolic deastatination in animal models, thereby realizing *in vitro* and *in vivo* evaluations of targeted functionalities.^26^–^28^ Wilbur *et al.* developed a variety of ^211^At-labeling reagents for use with biofunctional molecules, such as biotin,^29^ streptavidin,^30^ anti-renal cell carcinoma antibody A6H F(ab′)_2_,^31^ and anti-CD45 antibody.^32^ Li *et al.* recently reported the use of α-emitting locoregional therapy in human epidermal growth factor receptor 2 (HER2)-expressing gastric cancer in a xenograft mouse model. The stanylphenyl group was used to prepare the ^211^At-labeled trastuzumab.^33^ These studies prepared the ^211^At-labeled molecules by introducing prosthetic groups onto biofunctional molecules using *N*-succinimidyl ester^34^ or isothiocyanate derivatives,^35^ which are reactive toward amino groups on lysine residue side chains, or a maleimide derivative, which is reactive toward thiols on cysteine residue side chains.^36^ Conjugation was achieved over long reaction times to ensure a high conversion rate, and extra procedures involving reductants were needed to cleave the disulfide bond of antibodies.^22^ Efficient methods for introducing prosthetic groups into biomolecules for ^211^At-labeling using highly reactive and/or chemoselective conjugation approaches, such as click chemistry, have not yet been reported. α-Emission therapies using ^211^At-labeled molecular targeting agents may be extended by developing efficient methods of preparing ^211^At-labeled molecules.

**Scheme 1:**
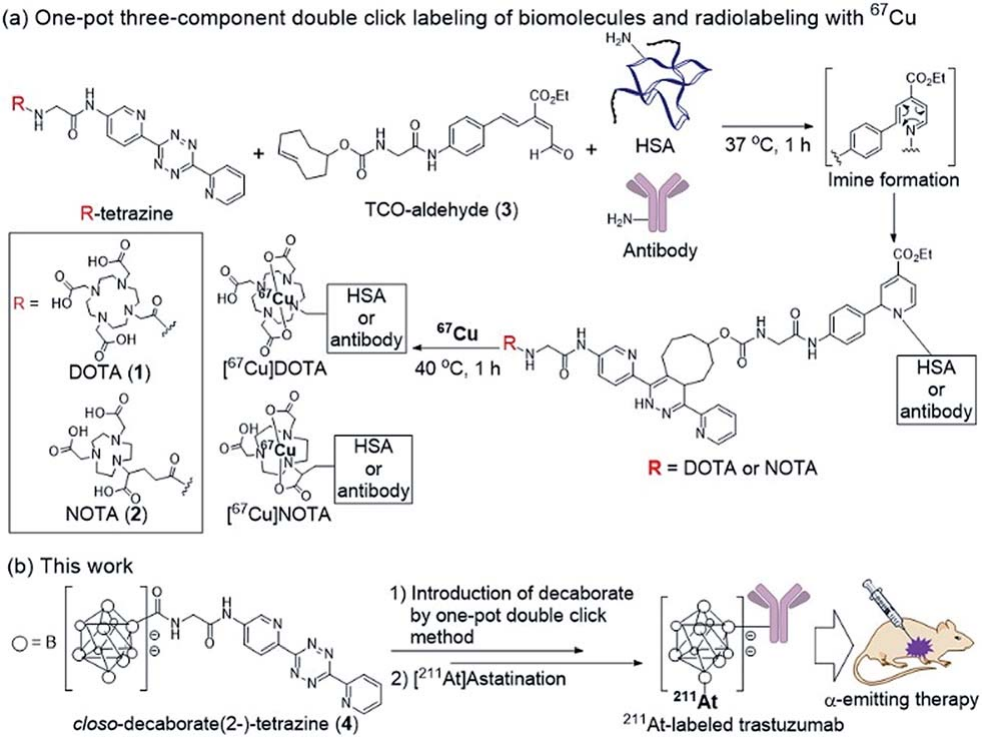
(a) Radiolabeling of human serum albumin (HSA) and an antibody using ^67^Cu *via* the one-pot three-component double-click method. (b) Design of the *closo*-decaborate(2-)-tetrazine probe and one-pot three-component double-click production of the ^211^At-labeled trastuzumab. Application to an α-emitting cancer therapeutic in a xenograft mouse model. DOTA: 1,4,7,10-tetraazadodecane-1,4,7,10-tetraacetic acid; NOTA: 1,4,7-triazacyclononane-1,4,7-triacetic acid; TCO: *trans*-cyclooctene.

Our group recently developed a facile method of radiolabeling amino groups on biomolecules using a “one-pot three-component double-click labeling” based on two types of click reactions in combination: a RIKEN click reaction, 6π-azaelectrocyclization,^37^–^42^ was combined with tetrazine ligation^43^ (Scheme 1).^44^ According to this labeling method, simply by mixing the DOTA (or NOTA)-tetrazine **1** (or **2**) as metal chelators and the TCO-aldehyde **3** (DOTA: 1,4,7,10-tetraazadodecane-1,4,7,10-tetraacetic acid, NOTA: 1,4,7-triazacyclononane-1,4,7-triacetic acid, tetrazine: 3,6-di-(2-pyridyl)-*s*-tetrazine, TCO: *trans*-cyclooctene) with biomolecules, such as proteins and antibodies, under mild conditions introduced DOTA (or NOTA) to biomolecules without loss of their bioactivities (Scheme 1a). Subsequent treatment of DOTA (or NOTA)-introduced biomolecules with ^67^Cu, which is an attractive radionuclide due to its diagnostic and therapeutic properties, efficiently produced ^67^Cu-labeled biomolecules. This radiolabeling method required only Amicon filtration for purification; thus, time-consuming purification by HPLC was not necessary. Note that based on our one-pot three-component double-click labeling, a variety of prosthetic radiolabeling groups could be introduced onto the amino groups of biomolecules without inhibiting their native functions.

The clinical use of ^211^At-labeled molecules would require methods of efficiently producing ^211^At. To date, only one report has addressed this obstacle.^45^ We envisioned that the world-class cyclotron at RIKEN, Japan, could be used to produce radioactive ^211^At in sufficient GBq units to reproducibly prepare α-emission cancer therapies for use in humans. In this context, we designed and synthesized a tetrazine probe **4** having *closo*-decaborate(2-) as the prosthetic group^46^ for binding ^211^At. We developed a facile non-invasive synthetic method of preparing ^211^At-labeled trastuzumab as an α-emitting therapeutic HER2-recognizing antibody using a double-click labeling strategy (Scheme 1b). Intratumor injection was found to provide excellent α-emitting therapeutic efficacy of the ^211^At-labeled trastuzumab against HER2-positive epidermoid cancer in a xenograft mouse model.

## Results and discussion

### Synthesis of the decaborate-tetrazine **4**

**Scheme 2:**
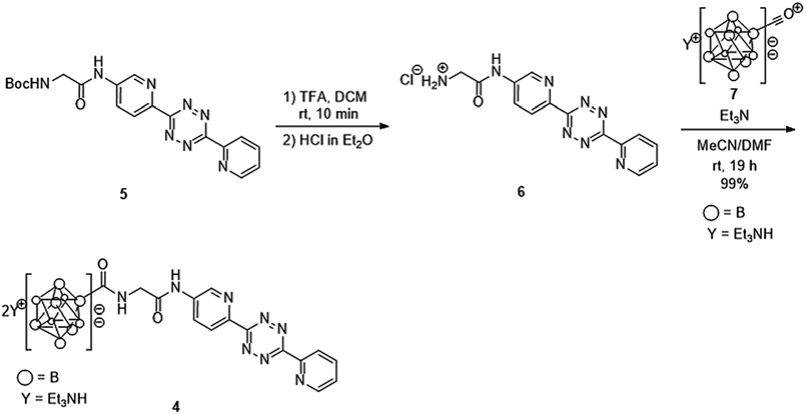
Synthesis of the *closo*-decaborate-tetrazine **4**. TFA = trifluoroacetic acid, DCM = dichloromethane, DMF = *N*,*N*-dimethylformamide.

We previously developed a concise method of labeling biofunctional molecules with DOTA and NOTA as conventional metal chelators without reducing their bioactivities using a one-pot three-component double-click method. Subsequent treatment of the DOTA/NOTA-labeled molecules with ^67^Cu enabled the synthesis of ^67^Cu-labeled biomolecules.^44^ This double-click method was applied to ^211^At-labeling by designing a tetrazine probe **4** bearing *closo*-decaborate(2-), which is stable *in vivo* against deastatination^47^ (Scheme 2). According to our previous synthesis,^44^ the glycine-labeled tetrazine **5** was deprotected to give the amine **6** as a hydrochloride salt. **6** was then coupled with the oxocarbenium derivative of *closo*-decaborate(2-), **7**, prepared according to the procedure reported by Wilbur,^24^ and the decaborate-tetrazine **4** was obtained in good yield over 2 steps.

### Synthesis of decaborate-trastuzumab *via* a one-pot three-component double-click reaction

**Scheme 3:**
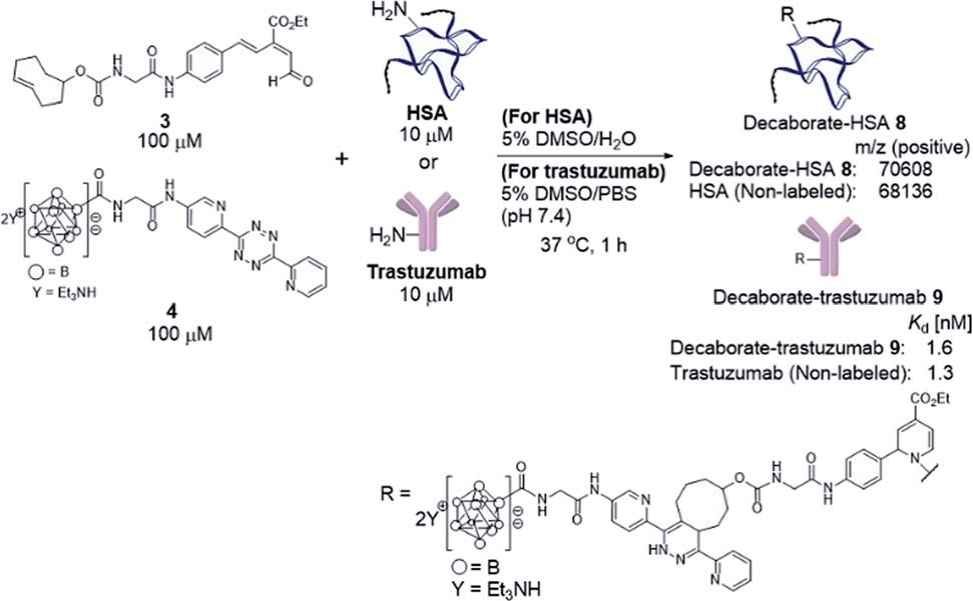
Preparation of the decaborate-albumin **8** and trastuzumab **9***via* the one-pot three-component double-click labeling method. Purification was performed by ultrafiltration using an Amicon filter, 50 000 molecular weight. Mass analysis of the decaborate-HSA **8** by MALDI-TOF. Dissociation constants (*K*_d_) of the decaborate-trastuzumab **9** measured by the QCM method. DMSO = dimethylsulfoxide. QCM = quartz-crystal microbalance.

With the decaborate-tetrazine **4** and TCO-aldehyde **3** in hand, we next examined the introduction of the decaborate moiety to biomolecules using a one-pot three-component double-click strategy. A model protein, HSA (human serum albumin), was modified with the decaborate moiety in the presence of 100 μM **3**, 100 μM **4**, and 10 μM HSA in a 5% DMSO aqueous solution at 37 °C for 1 h (Scheme 3). In our previous investigation of one-pot double click labeling, we established the conditions, *e.g.*, reagent concentrations, in which the tetrazine ligation between TCO-aldehyde **3** and tetrazine was completed within a few minutes, while the RIKEN click reaction proceeds over an hour to label a few lysines. In order to further ensure the completion of the tetrazine ligation, we initially mixed the TCO-aldehyde **3** and decaborate-tetrazine **4** prior to adding the biomolecules in one-pot manner. The reaction mixture was briefly purified by Amicon ultrafiltration to remove unreacted labeling probes. MALDI-TOF mass spectroscopic analysis of the obtained decaborate-HSA **8** revealed that an average of 2.3 decaborate moieties were attached to each HSA (Scheme 3). The number of attached molecules (a **3** + **4** molecule underwent a 1065 MW increase) was determined in comparison to the intact HSA molecular weight. Under the conditions used to label HSA, trastuzumab, which is commercially available and a promising anti-cancer antibody drug, was treated with the TCO-aldehyde **3** and decaborate-tetrazine **4** to prepare the decaborate-labeled trastuzumab **9** in a one-pot double click manner (Scheme 3). Although the exact molecular weight of the decaborate-trastuzumab **9** could not be detected using MALDI-TOF mass analysis (presumably due to the highly hydrophobic nature of the decaborate moiety), the number of prosthetic group introduced was found to be similar to the number introduced into the decaborate-HSA **8**, as reported previously.^44^ This was confirmed using radiolabeling experiments, as discussed below.

The recognition activity of **9** was investigated by measuring the dissociation constant (*K*_d_) of **9** using the quartz crystal microbalance (QCM) method (as described in the ESI†). As shown in Scheme 3, the dissociation constant of **9** was determined to be 1.6 nM, comparable to the dissociation constant of the non-labeled trastuzumab, 1.3 nM. The double click method introduced a decaborate moiety into trastuzumab without diminishing its binding affinity.

### Preparation of ^211^At-labeled trastuzumab

With the biologically functionally intact decaborate-trastuzumab **9** in hand, we next optimized the astatination of **9** under mild conditions. The astatination of **9** was conducted by treating solutions of **9** (at various concentrations) with Na[^211^At] in the presence of chloramine T as an oxidant^24^,^48^ over 5 min at room temperature (Table 1). We optimized the ^211^At labeling by treating 10 μM **9** with 5.8 MBq Na[^211^At] in phosphate buffer saline (PBS). The ^211^At-labeled trastuzumab **10** was successfully obtained in 78% RCY with a specific activity of 0.050 MBq μg^–1^ (Table 1, entry 1). The absence of non-specific binding between ^211^At and trastuzumab was confirmed by mixing a 10 μM PBS solution of the intact trastuzumab with Na[^211^At], 5.2 MBq (Table 1, entry 2). After ultrafiltration of the reaction mixture, most of the loaded Na[^211^At] was washed away, and very weak radioactivity from ^211^At, attributed to residual Na[^211^At], was observed in the purified trastuzumab. To reduce the administered quantity and to maximize the radiotherapeutic efficacy of the ^211^At-labeled trastuzumab in *in vivo* experiments (with an eye for future human applications), we prepared the ^211^At-labeled trastuzumab **10** with a higher specific activity by increasing the radioactivity of Na[^211^At] and by reducing the loading of trastuzumab. The labeling was performed using 1 μM **9** in 0.05% PBS-T and Na[^211^At], 75 MBq, in PBS to furnish **10** with a specific activity of 1.7 MBq μg^–1^ in 49% RCY (Table 1, entry 3). The potential loss of antigen recognition activity in the ^211^At-labeled trastuzumab with a high specific activity was assessed by measuring the dissociation constant *K*_d_ of the obtained **10**. This value was found to be 1.0 nM, indicating no impairment to the affinity. Reacting 0.1 μM **9** with Na[^211^At], 104 MBq, in PBS, provided **10** in 30% RCY with a very high specific activity of 15 MBq μg^–1^ (Table 1, entry 4). In this case, the dissociation constant of **10** could not be measured exactly due to the very low concentration of the antibody in the product. We also conducted a TLC analysis of **10** after Amicon ultrafiltration to confirm the formation of the ^211^At-trastuzumab complex (see Table S1 in the ESI†).

**Table 1:** Optimization of [^211^At] astatination of the decaborate-trastuzumab **9**

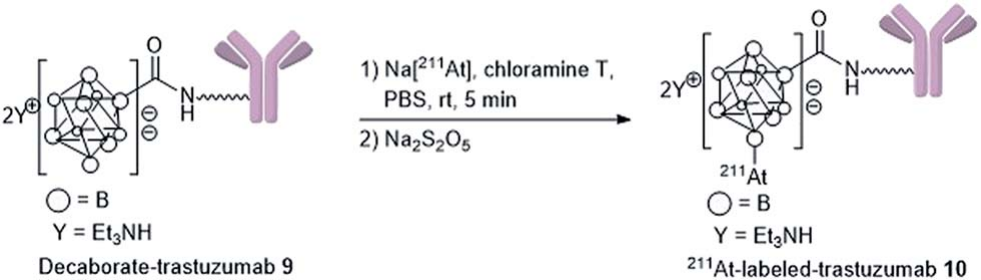					
Entry	Conc. of 9 (μM)	Na[^211^At] (MBq)	RCY (%)	Specific activity (MBq μg^–1^)	*K*_d_ ^*a*^ (nM)
1	10	5.8	78	0.050	—
2	10^*b*^	5.2	5	—	—
3	1	75	49	1.7	1.0
4	0.1	104	30	15	—^*c*^

^*a*^Dissociation constants were estimated using the QCM method.

^*b*^Instead of **9**, trastuzumab without the decaborate label was used as a negative control.

^*c*^The dissociation constant could not be measured accurately due to the very low amount of antibody present. Conc. = concentration. RCY = radiochemical yield.

**Fig. 1:**
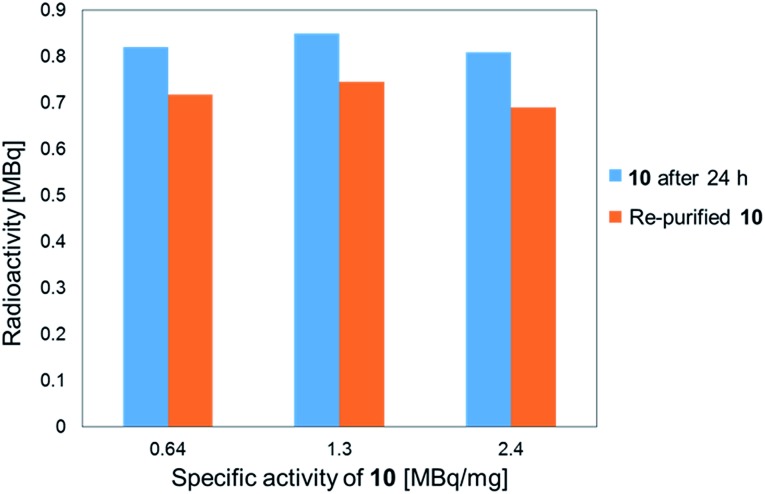
Stability analysis of the ^211^At-labeled trastuzumab **10**. The radioactivity of **10** and the specific activity was measured for each sample 24 h after sample preparation (light blue bar) and after re-purification of the samples (orange bar).

The stability of the ^211^At-labeled trastuzumab **10** was investigated by storing PBS solutions of **10** with different specific activities for 24 h at room temperature. The stability tests were conducted by preparing the ^211^At-labeled trastuzumab **10** with specific activities of 0.64, 1.3, or 2.4 MBq μg^–1^ under the following labeling conditions: 1, 0.5, or 0.25 μM **9** were treated with Na[^211^At], 22 MBq, respectively. The stock solutions in PBS were subjected to ultrafiltration, and the radioactivities were measured. As shown in Fig. 1, the ^211^At-labeled trastuzumab **10** solutions prepared with specific activities of 0.64, 1.3, and 2.4 MBq μg^–1^ displayed radioactive ^211^At corresponding to 0.82, 0.85, and 0.81 MBq, respectively, after 24 h and before Amicon ultrafiltration. After re-purification by Amicon ultrafiltration, ^211^At solutions of 0.72, 0.75, and 0.69 MBq were detected in the re-purified **10**; thus, the ^211^At-labeled trastuzumab synthesized *via* double clicks was robust, and degradation to small peptide fragments under proximal α-ray radiation produced by ^211^At was minimal.

### Biodistribution and α-emission therapeutic efficacy of ^211^At-labeled trastuzumab

In addition to efficiently synthesizing ^211^At-labeled trastuzumab, **10**, without degrading the HER2 recognition activity, we evaluated the biological function as an α-emitting cancer therapy *in vivo* using tumor-bearing mice. We first performed a biodistribution study of the ^211^At-labeled trastuzumab **10** in a xenograft model mouse using A431 human epidermoid carcinoma cells as HER2-positive cancer cells to examine the specific accumulation of **10** at the tumor. We previously reported the PET imaging of trastuzumab labeled with ^64^Cu-DOTA in the A431 xenografted mouse model, the same model as used in this study.^49^ The HER2 specific accumulation of ^64^Cu-labeled trastuzumab (20% ID/g of trastuzumab in tumor) was clearly visualized after intravenous injection.

**Fig. 2:**
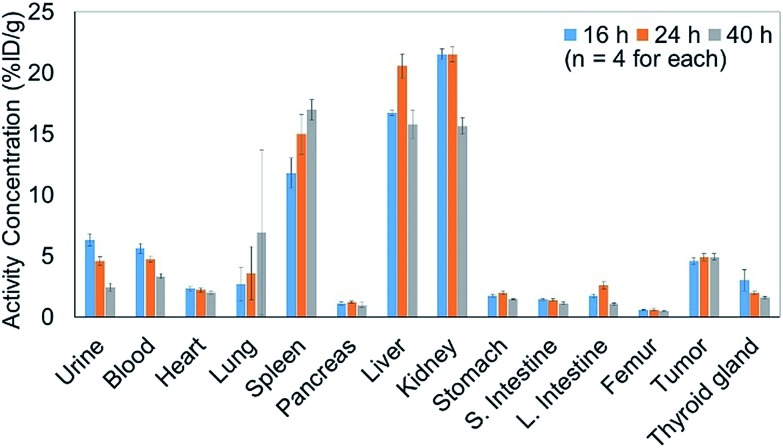
Concentration of the ^211^At-labeled trastuzumab **10** in each organ of the A431 cell xenograft mouse model. A 150 μL 0.05% PBS-T solution containing 20 μg **10** labeled with ^211^At (0.60 MBq) was intravenously injected into the A431 xenograft mice (*n* = 4). The radioactivity of each organ was measured after dissection at 16 h (light blue bar), 24 h (orange bar), or 40 h (gray bar) post injection.

We therefore prepared **10** with a specific activity of 0.030 MBq μg^–1^ after adjusting the appropriate volume of 0.05% Tween 20 – containing PBS (PBS-T) solution for injection into the mice (see the ESI† for protocol details). Next, 150 μL of a 0.05% PBS-T solution containing 20 μg **10** labeled with ^211^At, 0.60 MBq, was intravenously injected into each tumor-bearing mouse. The radioactivity in each organ dissected 16, 24, and 40 h after intravenous injection was measured, and high radioactivity was detected only in the liver and kidney, and the selective accumulation of **10** was not observed in the tumor (Fig. 2). Specific accumulation in the tumor did not improve upon intravenously injecting a reduced amount of **10** with a higher specific activity (see Fig. S3 in the ESI†).

Since the stability of the “RIKEN click” linkage was concerned in serum after intravenous injection, we performed the PET imaging (intravenous injection in A431 xenografted mouse) of ^64^Cu-DOTA labeled trastuzumab, which was similarly prepared *via* one-pot double click reaction, using DOTA-tetrazine and TCO-aldehyde **3** (see Fig. S5 in the ESI†). The PET image of ^64^Cu-labeled trastuzumab in Fig. S5a† clearly showed the accumulation in A431 tumor. In addition, the biodistribution studies after 2 days (Fig. S5b†) found sufficient radioactivity in tumor and blood, and less radioactivity in kidney in comparison with those obtained by intravenously injected ^211^At-labeled trastuzumab (Fig. 2). Alternatively, biodistribution experiments using the [^211^At]decaborate moiety provided similar results to ^211^At-labeled trastuzumab (Fig. S4 in the ESI†), *i.e.*, uptake in the liver followed by clearance through the kidneys. These results indicate that the low tumor uptake under intravenous injection of antibody is not due to the instability of “RIKEN click” linkage in serum, but rather introduction of a large hydrophobic decaborate moiety into the antibody significantly altered the *in vivo* kinetics. Namely, the hydrophobic trastuzumab conjugate could be captured and degradated by the liver for the renal clearance.

**Fig. 3:**
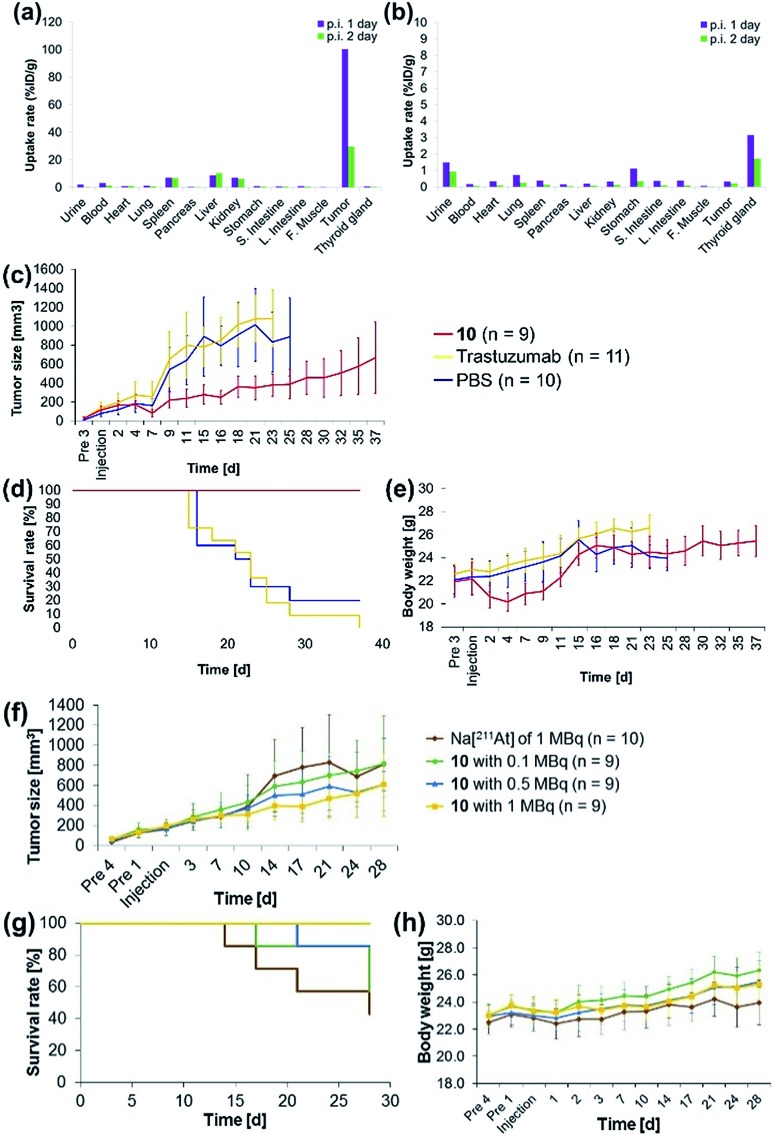
Concentrations of (a) the ^211^At-labeled trastuzumab **10** or (b) a 0.05% PBS-T solution of Na[^211^At] in each organ of the A431 cells xenograft mouse. (a) 6.3 μg **10** labeled with ^211^At (1.4 MBq) in 5 μL 0.05% PBS-T or (b) ^211^At (1.0 MBq) in 5 μL PBS were administered to the A431 xenograft mouse by intratumor injection. The radioactivity of each organ was measured after dissection 1 day (purple bar) or 2 days (green bar) post-injection. Therapeutic efficacies of the α-emitting **10** (red line), trastuzumab (non-labeled, yellow line), or PBS (blue line) after intratumor injection: (c) tumor size, (d) survival curve, and (e) body weight of the xenograft mice after intratumor injection. Dose dependence of the α-emitting therapeutic efficacy of ^211^At after intratumor injection of Na[^211^At] (brown line), **10** with 0.1 MBq (green line), 0.5 MBq (blue line), and 1 MBq (yellow line). (f) Time course analysis of the tumor size. (g) Survival curves of the tumor-bearing mice. (h) Body weight of the tumor-bearing mice after intratumor injection. The tumor size was measured in tumor xenograft mice with tumor sizes exceeding a volume (*V*) of 1200 mm^3^, calculated using the formula *V* = *Rr*^2^/2. Here, *R* and *r* indicate the long and short axes of the tumor in the xenograft mouse, respectively.

The antibody valency of the ^211^At-labeled trastuzumab **10** was exploited to achieve selective therapeutic effects in tumor regions by applying intratumor injections of **10** to the tumor-bearing mice. Intratumor injections of **10** with a specific activity of 0.41 MBq μg^–1^ were conducted by preparing 5 μL of a 0.05% PBS-T solution containing 6.3 μg **10** labeled with ^211^At, 1.4 MBq, and administering this solution to each tumor xenograft mouse. The radioactivity in each organ of the mouse dissected after 1 day post-injection was measured, revealing that most of the administered **10** remained in the tumor (Fig. 3a). Furthermore, even after 2 days post-injection, a sufficient quantity of the radioactive **10** could be observed in the tumor. By contrast, administration of Na[^211^At], 1.0 MBq, in 5 μL PBS to the tumors of xenograft mice *via* intratumor injection did not result in ^211^At radioactivity in the tumor, apparently because the salt leached out of the blood vessels (Fig. 3b). Although a relatively high level of ^211^At radioactivity was detected in the thyroid gland, in agreement with the literature,^50^,^51^ most ^211^At administered had been excreted from the bodies of the mice after 1 day post-injection. Thus, even under intratumor injection conditions, the tumor-anchoring effects of the ^211^At by the antibody were notable, highlighting the utility of the ^211^At antibody conjugate.

Encouraged by the success of the specific accumulation of **10** in the tumor, we evaluated tumor growth in A431 cell xenograft mice after intratumoral injection of **10**. A 0.05% PBS-T solution of **10**, the same concentration as was synthesized for the biodistribution experiments, was directly administered to the tumors of A431 cell xenograft mice. The long and short axes of the tumors in the xenograft mice were measured and the size was calculated every couple of days according to Battelle Columbus Laboratories Protocol^52^ (Fig. 3c). In mice administered **10** labeled with ^211^At, 1.4 MBq, the average tumor size in the xenograft mice decreased 7 days post-injection, as indicated by the red line. Successive tumor measurements over one month revealed that tumor growth was inhibited by the administration of **10**. By contrast, the xenograft mice injected with non-labeled trastuzumab (6.3 μg per mouse) or PBS, used as negative controls, displayed no therapeutic effect, and the average tumor size in the xenograft mice increased dramatically (Fig. 3c, yellow and blue lines). The survival curve revealed that the mice administered **10** did not die (Fig. 3d, red line). The faster tumor growth observed in the two groups of mice to which non-labeled trastuzumab and PBS were administered revealed that most of the mice died by 25 days post-injection (Fig. 3d, yellow and blue lines). Trastuzumab (Herceptin) is a clinically used drug, which is available from Chugai Pharmaceutical Co., Ltd. According to regulated information from electronic Medicines Compendium (UK),^53^ 8 mg kg^–1^ body weight is suggested for the initial loading dose, and 6 mg kg^–1^ body weight for the maintenance dose at three-week intervals. When this therapeutically effective dose to human patient was converted to the xenografted mouse (calculated as 25 g of mouse weight), the 200 μg of initial loading and the 150 μg of maintenance dose of trastuzumab are required. In our experiments, only 6.3 μg of trastuzumab was initially administered and no maintenance injections were performed. Therefore, the therapeutic effects could not be observed without labeling with ^211^At.

Importantly, although a 9% body weight loss (relative to the initial weight) was observed 4 days post-injection in the mice administered **10**, the body weight recovered its initial value over the following 8 days. Serious toxicity associated with ^211^At was not, therefore, observed (Fig. 3e, see below for detailed discussion for the toxicity).

The α-emission therapeutic efficacy of ^211^At was assessed using **10** by investigating the dose-dependent therapeutic effects of ^211^At. We prepared a series of **10** labeled with ^211^At, 1, 0.5, or 0.1 MBq using 6.3 μg antibody in 5 μL 0.05% PBS-T. These solutions were prepared from a 0.05% PBS-T solution of **10** labeled with ^211^At of 63 MBq (see the ESI† for protocol details). Intratumoral injection of 5 μL of each 0.05% PBS-T solution containing the radioactive **10**, or containing Na[^211^At], 1 MBq, as a free ^211^At sample, was administered to A431 xenograft mice, and the tumor growth in each mouse was monitored daily over one month. Tumor growth in the three groups of the mice did not differ until 10 days post injection. After two weeks post-injection, the tumors grew more slowly in the mice administered higher doses of **10** (Fig. 3f, green, blue, and yellow lines). In the absence of trastuzumab as a cancer-targeting molecule, no radiotherapeutic effect was observed, even though Na[^211^At], 1 MBq, should have been sufficient to provide effective therapy (*vide infra*, Fig. 3f, brown line). The survival curve revealed that all mice in the group administered **10** labeled with ^211^At, 1 MBq, remained alive during the monitoring period (survival rate of 100%, Fig. 3g, yellow line). On the other hand, the first death was observed after 14 days post-injection among the mice administered Na[^211^At]. Initial deaths occurred 17 or 21 days post-injection among mice administered **10** labeled with ^211^At, 0.1 or 0.5 MBq, respectively (Fig. 3g, brown, green, and blue lines). After 28 days post-injection, survival rates reached 42% among mice administered Na[^211^At], 57% among mice administered **10** labeled with ^211^At, 0.1 MBq, and 85% among mice administered **10** labeled with ^211^At, 0.5 MBq (Fig. 3g, brown, green, and blue lines). The intratumoral injection of **10** labeled with ^211^At, 1 MBq, was found to be quite effective in prolonging survival in this mouse model.

## Conclusions

We described the facile synthesis of ^211^At-labeled trastuzumab, a potentially useful α-emitting cancer-targeting therapy, based on a one-pot three-component double-click labeling strategy. The ^211^At-labeled trastuzumab was prepared for *in vivo* use by employing *closo*-decaborate as a prosthetic group to create a stable complex with halogens, including astatine, and by developing the *closo*-decaborate-tetrazine **4**, synthesized in only two steps from the intermediate **5**, as reported previously, as a key labeling probe for the one-pot double-click labeling reaction. Using the decaborate-tetrazine **4** and TCO-aldehyde **3** as the RIKEN click probes and conditions under which an average of 2.3 decaborate moieties were attached to each HSA model protein, trastuzumab as an anti-HER2 antibody could be readily modified with decaborate in a little over 1 h using Amicon ultrafiltration. The decaborate-trastuzumab **9** was efficiently labeled with ^211^At within 5 min and without reducing the antigen recognition activity of the antibody. We developed the ^211^At-labeled trastuzumab **10** with a high specific activity (15 MBq μg^–1^) using highly radioactive ^211^At, >100 MBq, produced at the RIKEN Nishina Center.

Intratumor injection revealed that the ^211^At-labeled trastuzumab **10** specifically accumulated in the A431 tumor cells of a xenograft mouse model. The therapeutic efficacy of this construct *via* proximal α-emission of ^211^At was found to be high, especially upon administration of **10** labeled with ^211^At, >1 MBq. Significantly, therapeutic effects were observed even in the presence of small quantities of the antibody (6 μg antibody per mouse).

The toxicity by astatine radioactivity under the experimental conditions was not significant or even negligible. Hasegawa and co-workers recently reported that the intravenous injection of 0.5 MBq of ^211^At-labeled trastuzumab caused the temporal decrease in the number of leukocytes and slightly reduced the body weight.^33^ Our intratumor injection date cannot simply be compared with those of the intravenous injection, but the toxicity caused by intratumoral injection of 1 MBq antibody is less than that by 0.5 MBq of intravenous injection. In fact, we cannot observe the significant decrease in body weight when injected 1 MBq of antibody (Fig. 3h). We found the slight reduction of the body weight when injected the 1.4 MBq of antibody (Fig. 3e), but that is temporal, and the weight has recovered and kept normal after 2 weeks.

Finally, we found that the [^211^At] decaborate complex was not appropriate as an antibody-based therapeutic delivered by intravenous injection because the notable hydrophobic properties of the labeling agent may have altered the native biodistribution of the antibody. Intratumor injection of the ^211^At-labeled antibody may be performed efficiently after diagnosis, *e.g.*, by positron emission tomography. Our ^211^At-labeling strategy should enable access to antibodies as well as to a variety of cancer-targeting molecules, thereby providing one of the most practical ^211^At-labeling methods for developing molecular cancer radiotherapeutics, especially against micrometastases, blood cancer, or disseminated cancer cells, which are easily accessible from the blood vessels. In fact, ^211^At-labeled antibodies have recently been tested for the clinical trials.^54^ In combination with the pretargeted radioimmunotherapy,^55^ future translational research of the targeted α-therapy becomes more significant.

## Experimental

All commercially available reagents were used without further purification. Distilled water was purchased from Nacalai Tesque. All organic solvents were purchased from Wako Pure Chemicals Industries. All animal procedures were performed in accordance with the Guidelines for Care and Use of Laboratory Animals of RIKEN and approved by the Animal Ethics Committee of RIKEN (H29-2-103).

### One-pot three-component double-click reactions to attach decaborate to HSA (**8**)

DMSO solutions of the decaborate-tetrazine **4** (4 × 10^–3^ M, 10 μL) and TCO-aldehyde **3** (4 × 10^–3^ M, 10 μL) were mixed in distilled water (180 μL). To the solution was added an aqueous solution of HSA (2 × 10^–5^ M, 200 μL). The solution was heated to 37 °C. After 1 h, the reaction mixture was transferred into an Amicon molecular weight 10k filtration unit and centrifuged under 14 000 × *g* for 10 min. To the filter was added distilled water (400 μL), and the solution was centrifuged under 14 000 × *g* for 10 min. This wash was repeated 3 more times. The residue on the filter was collected to give **8**. The aqueous solution of **8** was adjusted to a concentration of 2 × 10^–5^ M for use. Mass spectral analysis of the decaborate-HSA was performed to estimate the number of decaborate moieties attached to each HSA protein. MALDI-TOF MS (positive): *m*/*z* 70 608 (68 136 for non-labeled HSA).

### One-pot three-component double-click reaction to attach decaborate to trastuzumab (**9**)

DMSO solutions of the decaborate-tetrazine **4** (4 × 10^–3^ M, 10 μL) and TCO-aldehyde **3** (4 × 10^–3^ M, 10 μL) were mixed in PBS (180 μL). To the solution were added a PBS solution of trastuzumab (2 × 10^–5^ M, 200 μL). The solution was heated to 37 °C. After 1 h, the reaction mixture was transferred into an Amicon molecular weight 50 000 filtration unit and centrifuged under 14 000 × g for 5 min. To the filter was added distilled water (400 μL), and the solution was centrifuged under 14 000 × *g* for 5 min. This wash was repeated 3 more times. The residue on the filter was collected to give **9** as a PBS solution.

### General procedure for ^211^At-labeling decaborate-trastuzumab (**10**)

#### Table 1, entry 1

To a 2 × 10^–5^ M PBS solution of **9** (30 μL, 91 μg) were added Na[^211^At] (5.8 MBq) in water (30 μL) and 0.5 mg mL^–1^ chloramine T in water (6 μL). The reaction mixture was stored without stirring at room temperature for 5 min. After the addition of 0.5 mg mL^–1^ sodium pyrosulfite (Na_2_S_2_O_5_) in water (6 μL), the solution was transferred into an Amicon 50 000 filtration unit, 0.05% PBS-T was added to wash the reaction tube, and the solution was centrifuged under 14 000 × *g* for 5 min. Four hundred milliliters of 0.05% PBS-T were added, and the solution was centrifuged at 14 000 × *g* for 5 min. α-Ray doses of the residue on the filter and filtrate were measured using a germanium semiconductor detector. The product **10**, labeled with ^211^At (4.5 MBq, 78% RCY) and having a specific activity (0.050 MBq μg^–1^), was obtained in a 0.05% PBS-T solution.

#### Table 1, entry 3

Labeling was carried out according to the procedure described above using a 1.4 × 10^–5^ M PBS solution of **9** (10.2 μL, 21.7 μg), Na[^211^At] (75 MBq) in PBS (120 μL), 1.0 mg mL^–1^ chloramine T in water (30 μL), and 1.0 mg mL^–1^ sodium pyrosulfite (Na_2_S_2_O_5_) in water (30 μL). α-Ray doses of the residue on the filter and filtrate were measured using a germanium semiconductor detector. The product **10**, labeled with ^211^At (37 MBq, 49% RCY) and having a specific activity (1.7 MBq μg^–1^), was obtained in a 0.05% PBS-T solution.

#### Table 1, entry 4

Labeling was carried out using a 1.4 × 10^–5^ M PBS solution of **9** (0.96 μL, 2.0 μg), Na[^211^At] (104 MBq) in PBS (120 μL), 1.0 mg mL^–1^ chloramine T in water (39 μL), and 1.0 mg mL^–1^ sodium pyrosulfite (Na_2_S_2_O_5_) in water (39 μL). α-Ray doses of the residue on the filter and filtrate were measured using a germanium semiconductor detector. The product **10**, labeled with ^211^At (30 MBq, 30% RCY) and having a specific activity (15 MBq μg^–1^), was obtained in a 0.05% PBS-T solution.

## Conflicts of interest

There are no conflicts to declare.

## Supplementary Material

Supplementary information
