# Supplementary material for: 211At-labeled immunoconjugate via a one-pot three-component double click strategy: practical access to α-emission cancer radiotherapeutics
Source: Chem Sci. 2018 Dec 21;10(7):1936–44. doi: 10.1039/c8sc04747b (PMC6385556; doi:10.1039/c8sc04747b)
Supplement: Supplementary file 1 [file SC-010-C8SC04747B-s001.pdf]

Electronic Supplementary Material (ESI) for Chemical Science.
This journal is © The Royal Society of Chemistry 2018

Supplementary Information

^{211}At-Labeled immunoconjugate via a one-pot three-component double click strategy: Practical access to α-emission cancer radiotherapeutics

Katsumasa Fujiki, Yousuke Kanayama, Shinya Yano, Nozomi Sato, Takuya Yokokita, Peni Ahmadi, Yasuyoshi Watanabe, Hiromitsu Haba, and Katsunori Tanaka*

1. General methods **S3**

2. Synthesis of *closo*-decaborate-tetrazine, (4) **S4**

3. Synthesis of *closo*-decaborate oxocarbenium ion, (7) **S5**

4. ^{211}At production **S6**

5. Control experiment for ^{211}At-labeling using non-labeled trastuzumab (Table 1, entry 2) **S7**

6. TLC analysis of ^{211}At-labeled trastuzumab 10 **S8**

7. Stability analysis of ^{211}At-labeled trastuzumab 10 with different specific activities (Fig. 1) **S9**

8. General protocol for QCM analysis (Scheme 3 and Table 1, entry 3) **S10**

9. Biodistribution by intravenous injection in A431 cells xenograft mice **S11**

9-1 Biodistribution of **10** S11

9-2 Biodistribution of ^{211}At-labeled *closo*-decaborate for control experiment S13

10. Biodistribution and therapeutic efficacy of 10 by intratumor injection in A431 cells xenograft mice (Fig. 3) **S14**

11. PET imaging and biodistribution of ^{64}Cu-labeled trastuzumab prepared by the one-pot double click method **S15**

12. NMR spectra **S16**

closo-decaborate-tetrazine, (**4**) S16

closo-decaborate oxocarbenium ion, (**7**) S17

13. References S18

1. General methods

All commercially available reagents were used without further purification. All anhydrous solvents and *closo*-decaborane ($B_{10}H_{14}$, CAS: 17702-41-9) were purchased from Wako Pure Chemical Industries. Trastuzumab (Herceptin®) was purchased from Chugai Pharmaceutical Co., Ltd. Other commercially available reagents were purchased from Sigma-Aldrich. Silica gel (Silica gel 60, 0.015−0.040 mm) for column chromatography was used. For preparative TLC, PLC glass plate Silica gel 60 F_{254}, 0.5 or 1 mm (Merck-Millipore) was used. For radio-TLC analysis of ^{211}At-labeled trastuzumab **10**, TLC aluminium sheet Silica gel 60 F_{254}, 0.5 mm (Merck-Millipore) was used.

Mass spectra of decaborate-attached albumin **8** were obtained on a Bruker autoflex spectrometer® by matrix assisted laser desorption ionization (MALDI-TOF MS), using 2,5-dihydroxybenzoic acid (DHB) as a matrix.

The antibody concentrations of **9** in PBS and **10** in 0.05% PBS-T were measured using a Microvolume Spectrophotometer DS-11 (DeNovix).

2. Synthesis of *closo*-decaborate-tetrazine, (4)

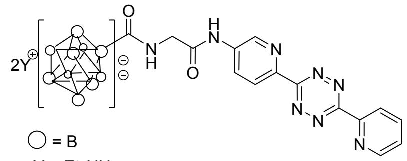

4

N-Boc-Gly-tetrazine **5** (1.5 mg, 0.0036 mmol) was dissolved in 50% TFA–containing DCM (0.26 mL), stirring for 10 min at room temperature. The reaction mixture was co-evaporated with toluene (3×). Then 1 N HCl in diethyl ether was added and co-evaporated with toluene. To the obtained material were added 70 μL dry MeCN solution of *closo*-decaborate oxocarbenium ion **7** (8.0 mg, 0.032 mmol) prepared according to the literature[1], dry DMF (20 μL), and Et_3N (3.0 μL, 0.021 mmol) under N_2 atmosphere. After stirring for 20 h at room temperature, EtOAc was added and the reaction mixture was stirred for 15 min, then filtered. The filtered crude material was purified by cosmosil $75C_{18}$-OPN (H_2O:MeCN 95:5 to 60:40) to afford **4** (2 mg, 87%) as light brown oil. ^{1}H NMR (400 MHz, CD_3CN): δ 8.58-8.50 (m, 1H), 8.00-7.84 (m, 5H), 7.44 (br, 1H), 3.72-3.69 (m, 2H), 3.22-3.12 (m, 12H), 1.22 (t, *J* = 6.8 Hz, 18H).

3. Synthesis of *closo*-decaborate oxocarbenium ion, (7)

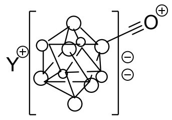

7

According to the literature[1], *closo*-decaborate oxocarbenium ion **7** (26 mg, 68%) was obtained. ^{1}H NMR (400 MHz, CD_3CN): δ 9.44 (br, 1H), 3.08-3.04 (m, 6H), 1.24 (t, *J* = 7.2 Hz, 9H).

4. ^{211}At production

The radioisotope of ^{211}At was produced in the nuclear reaction of ^{209}Bi(α,2n)^{211}At according to the procedure described[2,3]. A metallic ^{209}Bi target (chemical purity: >99.999%, typical thickness: 20 mg cm^{-2}) was prepared by a vacuum evaporation on an aluminum plate. The Bi target was placed at 15° incline with respect to the beam axis. The 29.4-MeV beam was delivered from the RIKEN AVF cyclotron; the beam energy on the center of the target surface was calculated to be 28.4 MeV with the SRIM-2013 program[4]. To obtain ^{211}At with a high radionuclidic purity, the beam energy was controlled to 28–29 MeV using electrostatic pickups to prevent the production of ^{210}At ($T_{1/2}$ = 8.1 h) which decays to a highly toxic α emitter ^{210}Po ($T_{1/2}$ = 138 d). The typical beam intensities were 5–10 μA. The target was cooled with the circulating water (1.5 L min^{-1}) and He gas (30 L min^{-1}) during the irradiation. The beam axis was continuously rotated in 3-mm diameter at 2 Hz to avoid a local heating of the ^{209}Bi target using a beam wobbling electromagnet on the beam line of the AVF cyclotron. The Bi targets were irradiated for several hours to produce maximum 1 GBq of ^{211}At.

After the irradiation, the ^{209}Bi target was placed on a copper tray in a quartz tube (28-mm i.d.×200-mm length) and heated up to 850°C using an electric furnace. ^{211}At sublimated from the target material was extracted from the quartz tube to a PFA tube (1-mm i.d.×1-m length) through a quartz capillary (2-mm i.d. ×130-mm length) with an O_2 gas flow at a flow rate of 20 mL min^{-1}. The PFA tube was cooled at –72°C in a mixture of dry ice and ethanol to collect the gaseous ^{211}At. After the distillation for 30 min at 850°C, the quartz capillary was removed from the quartz tube, and the inside of the quartz capillary and the PFA trap tube was washed with 100–200 μL of water to recover ^{211}At. The chemical yield of ^{211}At was determined by γ-ray spectrometry using a Ge detector. The radionuclidic purity was determined by α-particle spectrometry and γ-ray spectrometry using Si and Ge detectors, respectively.

The α-particle and γ-ray spectra of the purified ^{211}At are shown in Figs. a and b, respectively. The peaks on ^{211}At are only seen in the spectra. The chemical yields of ^{211}At were approximately 80%. The radionuclidic purity of the ^{211}At solution was >99.9% and the atomic ratio of ^{210}At/^{211}At was $< 1.0\times10^{-5}$ at the end of irradiation. Maximum 100 MBq of ^{211}At was used for the radiolabeling experiment.

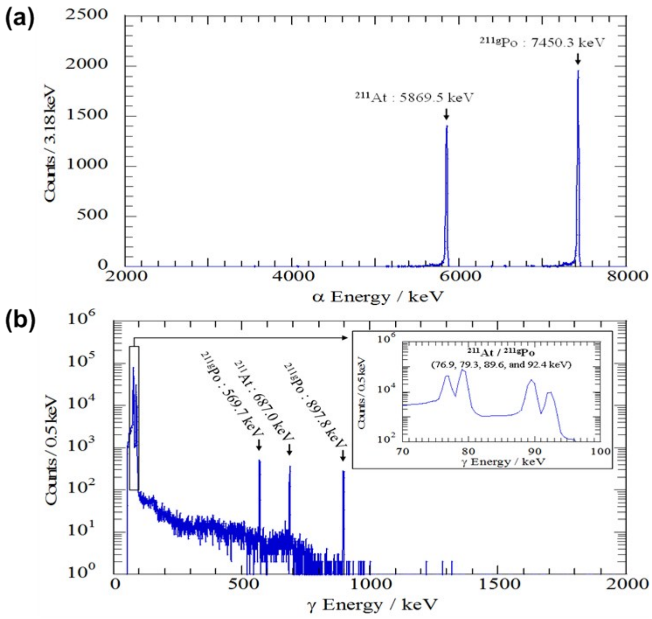

Fig. S1. Typical (a) α-particle and (b) γ-ray spectra of the purified ^{211}At from the ^{209}Bi target.

5. Control experiment for ^{211}At-labeling using non-labeled trastuzumab (Table 1, entry 2)

According to the general procedure, the labeling was carried out by using 2×10^{-5} M PBS solution of non-labeled trastuzumab (30 μL, 90 μg), Na[^{211}At] (5.2MBq) in PBS (30 μL), 1.0 mg/mL chloramine T in water (3 μL), and 1.0 mg/mL sodium pyrosulfite ($Na_2S_2O_5$) in water (3 μL). α-Ray doses of the residue on filter and the filtrate were measured by using germanium semiconductor detector and the residual Na[^{211}At] (0.27 MBq, 5% RCY) was detected on the filter.

6. TLC analysis of ^{211}At-labeled trastuzumab 10

According to the general procedure, the labeling was carried out by using 2×10^{-5} M PBS solution of **9** (0.6 µL, 1.8 µg), Na[^{211}At] (21.8 MBq) in PBS (120 µL), 1.0 mg/mL chloramine T in water (6.5 µL), and 1.0 mg/mL sodium pyrosulfite ($Na_2S_2O_5$) in water (6.5 µL). α-Ray doses of the residue on filter and the filtrate were measured by using germanium semiconductor detector and **10** labeled with ^{211}At (9.94 MBq, 46% RCY) with specific activity (5.5 MBq/µg) was obtained as 0.05% PBS-T solution. On TLC aluminium sheet, 1 µL of **10** in 0.05% PBS-T was spotted and the TLC plate was run with $CHCl_3$:MeOH 3:1 (run with this solvent, R_f of trastuzumab and NaI whose hydrophilicity is similar to Na[^{211}At] were 0.0 and 0.67, corresponding to area 1 and 3 on the TLC sheet in Table. S1, respectively). The TLC sheet was cut and separated 4 pieces, then X-Ray doses of them was measured by using germanium semiconductor detector.

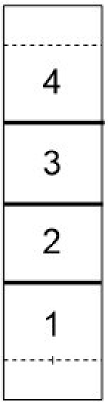

Area on TLC	X-ray dose [cps][a]	Distribution ratio [%]
1	383.5	95.7
2	6.60	1.6
3	2.90	0.7
4	7.73	1.9

Table. S1. TLC analysis of **10**. [a]cps = count per second.

7. Stability analysis of ^{211}At-labeled trastuzumab 10 with different specific activities (Fig. 1)

According to the general procedure, **10** with each specific activities was synthesized by treatment of 1.4×10^{-5} M PBS solution of **9** (6 μL, 18 μg), or 1.4×10^{-5} M PBS solution of **9** (3 μL, 9.1 μg), or 1.4×10^{-5} M PBS solution of **9** (1.5 μL, 4.5 μg) with Na[^{211}At] (22 MBq) in PBS (120 μL) and 1.0 mg/mL chloramine T in water (6.5 μL), and 1.0 mg/mL sodium pyrosulfite ($Na_2S_2O_5$) in water (6.5 μL) was used to quench the reactions for each. α-Ray doses of the residue on filter and the filtrate were measured by using germanium semiconductor detector and **10** labeled with ^{211}At (11.5 MBq, 53% RCY) with specific activity (0.64 MBq/μg), or **10** labeled with ^{211}At (11.8 MBq, 54% RCY) with specific activity (1.3 MBq/μg), or **10** labeled with ^{211}At (10.9 MBq, 50% RCY) with specific activity (2.4 MBq/μg) were obtained as 0.05% PBS-T solutions for each. Each samples were diluted with PBS (400 μL) and left at room temperature for 24 h. α-Ray doses of these samples were measured by using germanium semiconductor detector before and after amicon centrifuge under 14,000 ×g for 5 min.

8. General protocol for QCM analysis (Scheme 3 and Table 1, entry 3)

To evaluate the immunoreactivity, the dissociation constants (K_d) of ^{211}At-labeled and non-labeled trastuzumab were determined by using the QCM system Single-Q 0500 with the software (QCM1-2 and Q-up Analysis, AsOne, Japan) according to the manufacturer's protocol. In brief, a sensor chip was mounted on the bottom of the reaction well, and 500 µl of PBS solution was then added to the well. After loading of a 5 µl of antigen solution (0.2 µg/µl, recombinant human ErbB2/Her2 Fc chimera protein, 1129-ER, R&D Systems), decrease in quartz frequency was monitored to determine the binding amount of the antigen to the sensor surface. In this QCM system, 30 pg binding may cause the decrease in frequency of 1 Hz. When the decrease in frequency reached equilibrium, the solution was removed. Then the reaction well was rinsed with 500 µl of PBS 3 times and the stirrer was washed with ultra-pure water. And then 500 µl of PBS solution was added again to the well and a 5 µl of 5 % bovine serum albumin in PBS solution was loaded as a blocking agent. And then, to obtain saturation binding curve, the decrease in frequency was monitored for 5 to 6 times following repeated loading of each 5 µl of an antibody solution (0.05 µg/µl). Saturated binding amounts, ΔF [Hz], which calculated from the curve fitting of frequency reduction in QCM sensorgram were plotted as a function of each antibody concentrations [M]. The data were rearranged to the Scatchard plot where ΔF/concentration [Hz/M] was plotted as a function of ΔF [Hz]. The plot of ΔF/concentration versus ΔF was fitted to a straight line with a slope of $-1/K_D$.

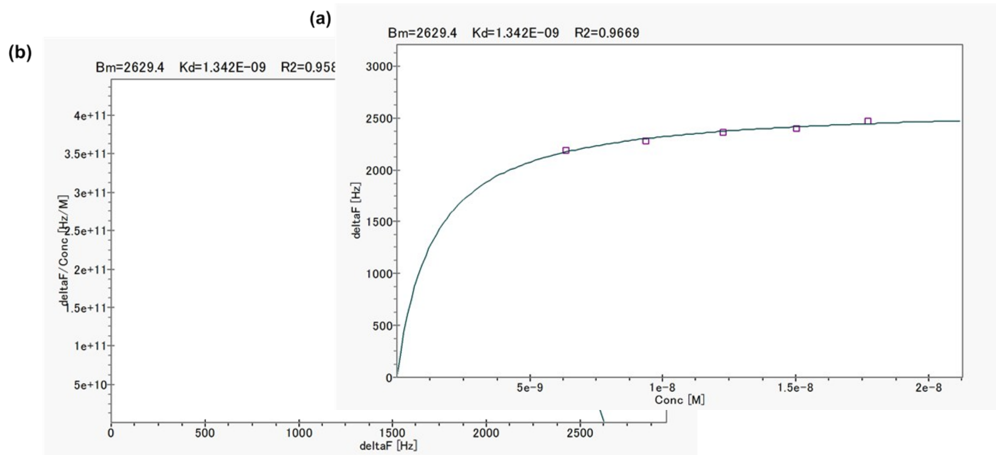

Fig. S2. (a) Typical examples of saturation binding plot (Michaelis-Menten) and (b) scathcard plot in QCM analysis of ^{211}At-labeled trastuzumab immunoreactivity.

9. Biodistribution by intravenous injection in A431 cells xenograft mice

9-1. Biodistribution of 10

(**Fig. 2**) According to the general procedure, the ^{211}At-labeling was carried out by using 2×10^{-5} M PBS solution of **9** (100 μL, 304 μg), Na[^{211}At] (69.8 MBq) in PBS (100 μL), 1.0 mg/mL chloramine T in water (13 μL), and 1.0 mg/mL sodium pyrosulfite ($Na_2S_2O_5$) in water (13 μL). α-Ray doses of the residue on filter and the filtrate were measured by using germanium semiconductor detector and **10** labeled with ^{211}At (27.2 MBq, 39% RCY) with specific activity (0.089 MBq/μg) was obtained as 0.05% PBS-T solution. The solution was diluted with PBS solution of trastuzumab, then, 150 μL 0.05% PBS-T solution of **10** (0.60 MBq, 20 μg) per mouse was administered to 12 mice of A431 cells xenograft mice in total by intravenous injection and γ-ray doses of each organs after dissection at 16, 24, and 40 h were measured by γ-ray counter.

(**Fig. S3**) According to the general procedure, the ^{211}At-labeling was carried out by using 1.4×10^{-5} M PBS solution of **9** (15 μL, 31.9 μg), Na[^{211}At] (87.4 MBq) in PBS (184 μL), 1.0 mg/mL chloramine T in water (50 μL), and 1.0 mg/mL sodium pyrosulfite ($Na_2S_2O_5$) in water (50 μL). α-Ray doses of the residue on filter and the filtrate were measured by using germanium semiconductor detector and **10** labeled with ^{211}At (49.4 MBq, 56% RCY) with specific activity (1.67 MBq/μg) was obtained as 0.05% PBS-T solution. The 4 μL 0.05% PBS-T solution of **10** (1.54 MBq, 0.92 μg) per mouse was administered to 12 mice of A431 cells xenograft mice in total by intravenous injection and γ-ray doses of each organs after dissection at 3, 24, and 48 h were measured by γ-ray counter.

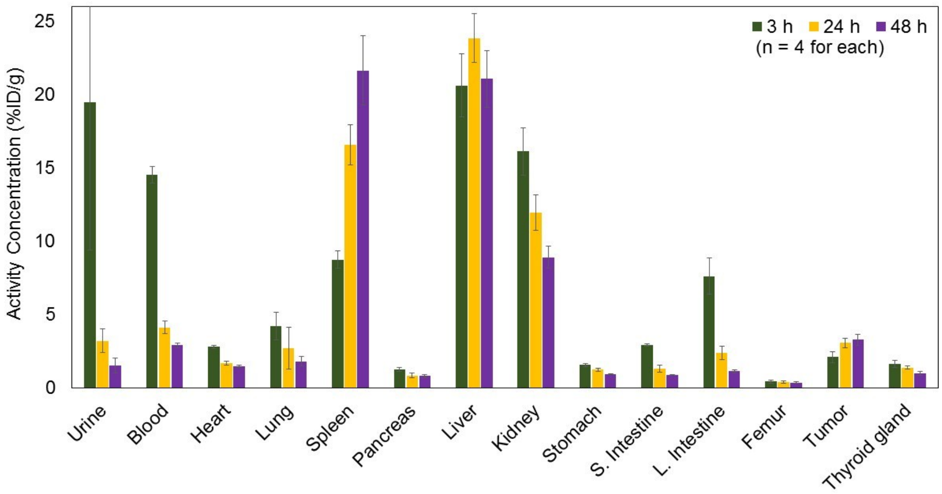

Fig. S3. Concentration of ^{211}At-labeled trastuzumab **10** in each organ of A431 xenografted mice. The 4 μL 0.05% PBS-T solution containing 0.92 μg of **10** labeled with ^{211}At, 1.54 MBq, was intravenously injected to the A431 xenograft mice (n = 4). Radioactivity of each organ was measured after dissection at 3 h (green bar), 24 h (yellow bar), or 48 h (purple bar) post injection.

9-2. Biodistribution of ^{211}At-labeled *closo*-decaborate for control experiment

To Na[^{211}At] (33 MBq) in PBS (150 μL) were added 2×10^{-5} M DMSO solution of decaborate-tetrazine (1.8 μL) and 1.0 mg/mL chloramine T in water (17 μL). After reaction for 5 min at room temperature, 1.0 mg/mL sodium pyrosulfite ($Na_2S_2O_5$) in water (17 μL) was added to stop the reaction. The solution was diluted with 680 μL of PBS, and then, 100 μL 0.2% DMSO–containing PBS solution of ^{211}At-labeled decaborate-tetrazine (3.9 MBq) per mouse was intravenously injected to 8 A431 xenografted mice.

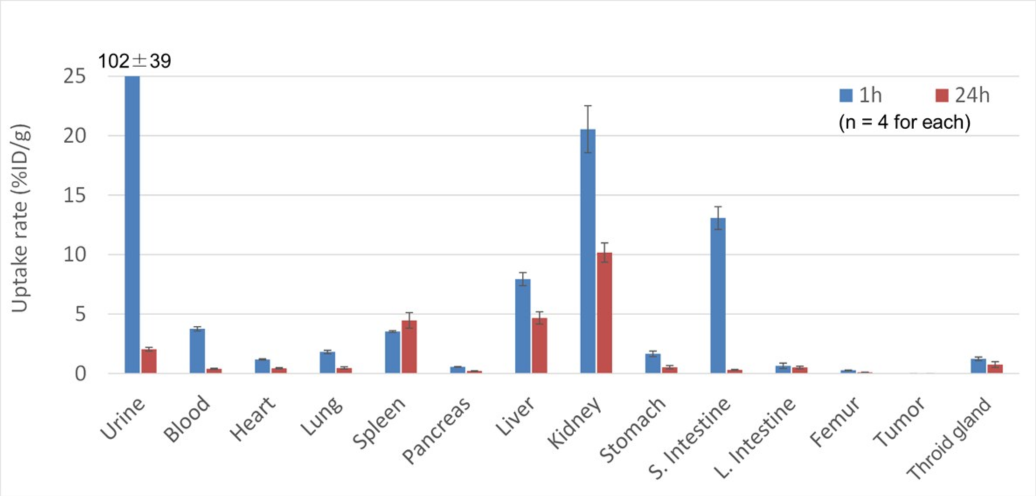

Fig. S4. Concentration of ^{211}At-labeled decatorate-tetrazine in each organ of A431 cells xenografted mice. (a) The 100 μL 0.2% DMSO–containing PBS solution of decaborate-tetrazine labeled with ^{211}At, 3.9 MBq, was intravenously injected to the A431 xenograft mice (n = 4). Radioactivity of each organ was measured after dissection at 1 h (blue bar) and 24 h (dark red bar) post injection. The 4 mice were dissected at each time points.

10. Biodistribution and therapeutic efficacy of 10 by intratumor injection in A431 cells xenograft mice

(Fig. 3a-e) According to the general procedure, the ^{211}At-labeling was carried out by using 1.4×10^{-5} M PBS solution of **9** (22 µL, 46 µg), Na[^{211}At] (34 MBq) in PBS (215 µL), 1.0 mg/mL chloramine T in water (17 µL), and 1.0 mg/mL sodium pyrosulfite ($Na_2S_2O_5$) in water (17 µL). α-Ray doses of the residue on filter and the filtrate were measured by using germanium semiconductor detector and **10** labeled with ^{211}At (19 MBq, 56% RCY) with specific activity (0.41 MBq/µg) was obtained as 0.05% PBS-T solution. By addition of trastuzumab in PBS to the solution, 61 µL of 0.05% PBS-T solution of **10** (19 MBq, 77 µg) was prepared and 5 µL of the solution per mouse was administered to 11 mice of A431 cells xenograft mice in total by intratumor injection. The trastuzumab solution in PBS (6.3µg, 5 µL per mouse) or PBS (5 µL per mouse) were administered to 11 mice of A431 cells xenografted mice in total by intratumoral injection.

(Fig. 3f-h) According to the general procedure, the ^{211}At-labeling was carried out by using 9.6×10^{-6} M PBS solution of **9** (26 µL, 38 µg), Na[^{211}At] (94 MBq) in PBS (200 µL), 1.0 mg/mL chloramine T in water (21 µL), and 1.0 mg/mL sodium pyrosulfite ($Na_2S_2O_5$) in water (21 µL). α-Ray doses of the residue on filter and the filtrate were measured by using germanium semiconductor detector and **10** labeled with ^{211}At (63 MBq, 68% RCY) with specific activity (1.7 MBq/µg) was obtained as 0.05% PBS-T solution. After addition of trastuzumab in PBS to the solution, 5 µL of each 0.05% PBS-T solutions of **10** (1 MBq, 6.3 µg), (0.5 MBq, 6.3 µg), and (0.1 MBq, 6.3 µg) per mouse was administered to 7 mice of A431 cells xenograft mice in total by intratumor injection.

11. PET imaging and biodistribution of ^{64}Cu-labeled trastuzumab prepared by the one-pot double click method.

Preparation of DOTA-trastuzumab by one-pot double-click method: According to our previous report (Sci. Rep. 2017.), the DOTA-trastuzumab was synthesized by reacting trastuzumab in PBS (2×10^{-5} M, 125 μL) and DMSO solutions of DOTA-tetrazine (2×10^{-3} M, 12.5 μL) and TCO-aldehyde **3** (4×10^{-3} M, 6.25 μL) in PBS (106 μL) at 37 °C for 1 h. After centrifugation under 14,000 ×g for 5 min using Amicon molecular weight 50,000 filtration unit, DOTA-trastuzumab (1.35×10^{-5} M, 160 μL) was obtained.

Preparation of ^{64}Cu-labeled trastuzumab: According to the reference,[5] ^{64}Cu-labeled trastuzumab was prepared by using PBS solution of DOTA-trastuzumab (60 μg) was added ^{64}Cu (580 MBq) in 0.1 M AcOH buffer (pH 6.5) and the solution was heated to 40 °C for 1 h. The reaction mixture was purified by Amicon molecular weight 50,000 filtration unit to afford ^{64}Cu-labeled trastuzumab (17 MBq, 3% RCY) with specific activity (0.29 MBq/μg).

The PBS solution of ^{64}Cu-labeled trastuzumab (12.8 MBq, 50 μg) was prepared and intravenously injected to A431 xenograft mouse. At 2 d post injection, PET imaging of ^{64}Cu-labeled trastuzumab and biodistribution analysis after dissection of the mouse were investigated.

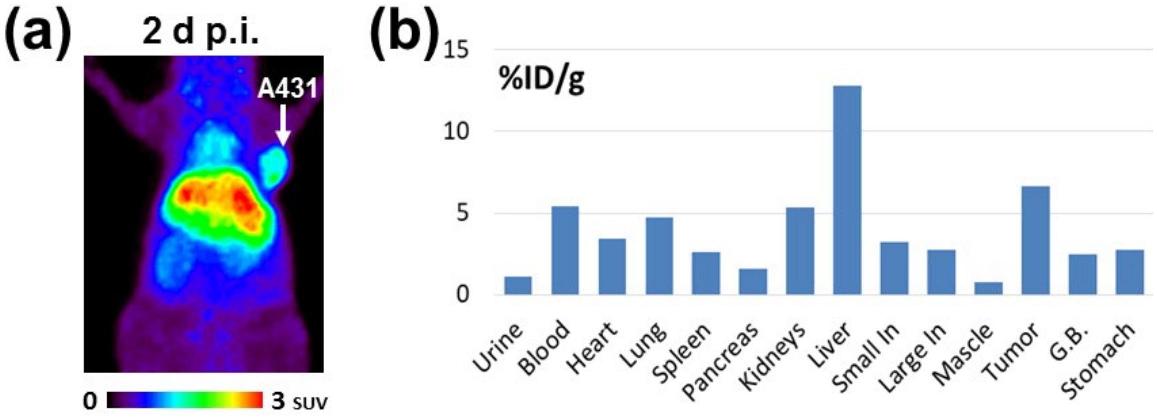

Fig. S5. (a) PET imaging and (b) biodistribution of ^{64}Cu-labeled trastuzumab in A431 xenograft mouse. (a) The uptake of ^{64}Cu-labeled trastuzumab is visualized by maximum-intensity projection. A431 tumor is pointed by the white arrow. (b) The biodistribution of ^{64}Cu-labeled trastuzumab was investigated by measuring γ-ray dose of each tissue after dissection of the mouse. G.B. = gallbladder.

12. NMR spectra

closo-decaborate-tetrazine, (**4**)

^{1}H NMR (400 MHz, CD_3CN)

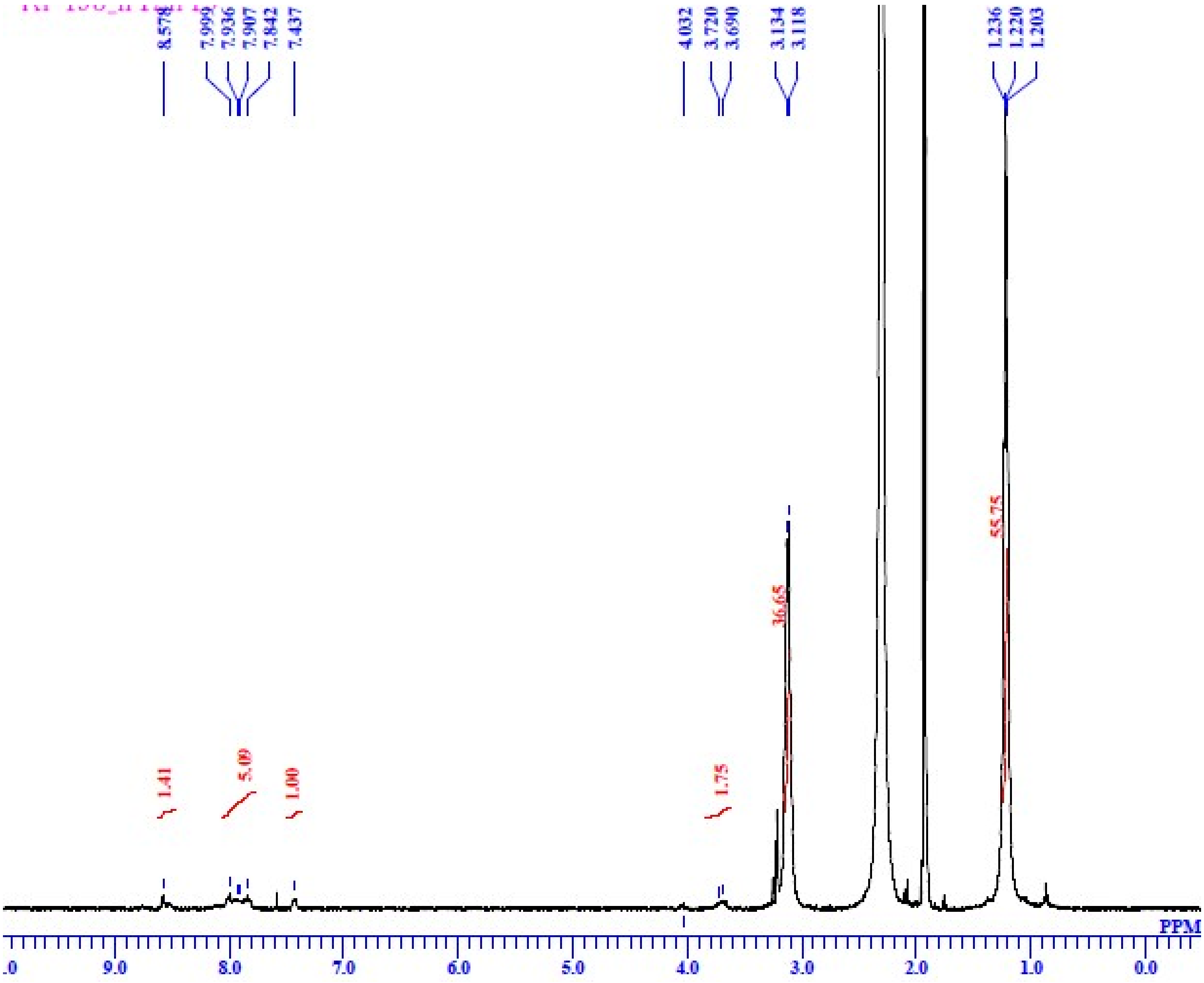

closo-decaborate oxocarbenium ion, (**7**)

^{1}H NMR (400 MHz, D_2O)

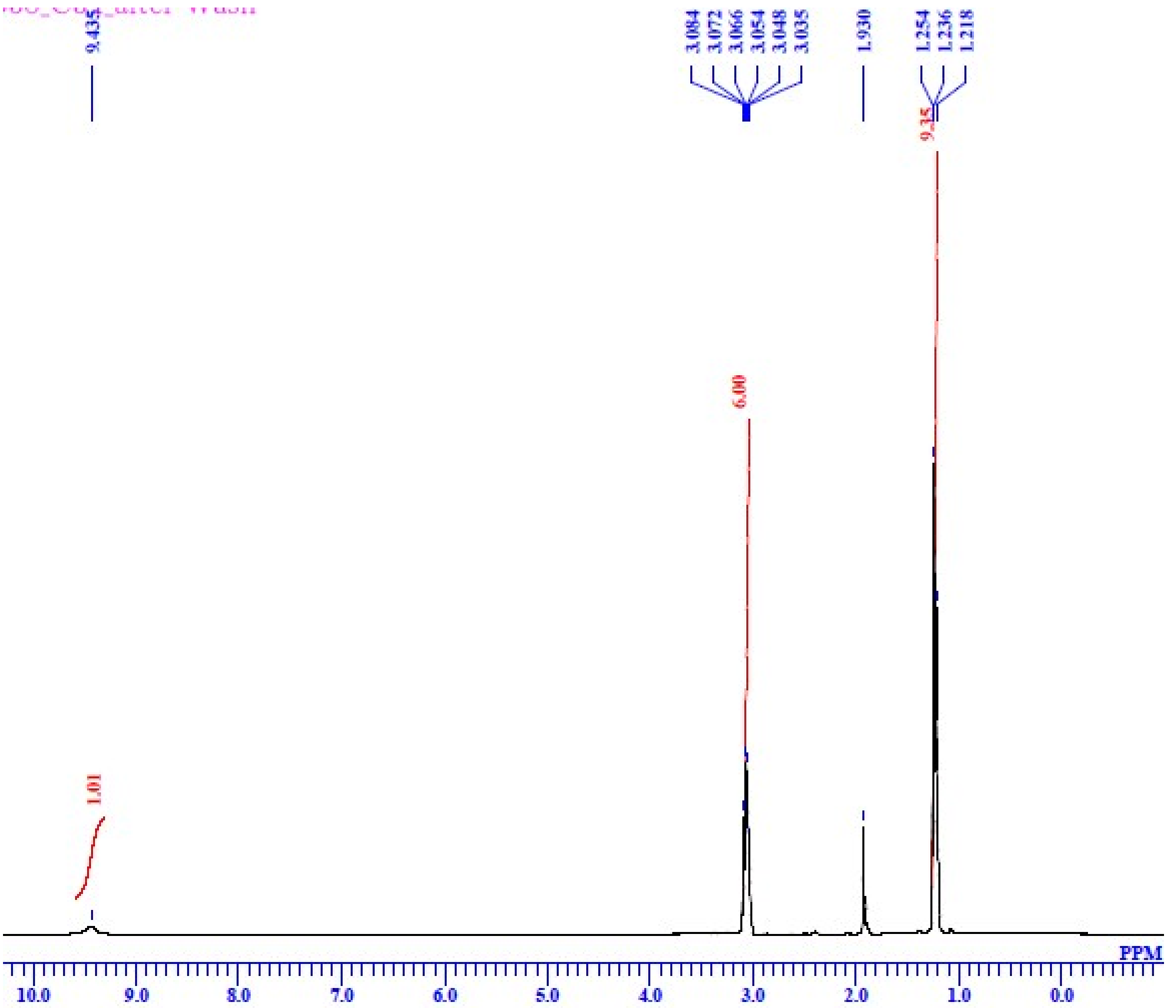

13. References

1. Wilbur, S. D., Chyan, M.-K., Hamlin, D. K., Perry, M. A. *Bioconjug. Chem.* **2009**, *20*, 591–602.

2. Sato, N., Yano, S., Toyoshima, A., Haba, H., Komori, Y., Shibata, S., Watanabe, K., Kaji, D., Takahashi, K., Matsumoto, M. *RIKEN Accel. Prog. Rep.* **2017**, *50*, 262.

3. Yano, S., Sato, N., Toyoshima, A., Haba, H., Komori, Y., Shibata, S., Takahashi, K., Matsumoto M. *RIKEN Accel. Prog. Rep.* **2017**, *50*, 263.

4. Ziegler, J. F., Ziegler, M. D., Biersack, J. P. *Nucl. Instrum. Meth. Phys. Res. B* **2010**, *268*, 1818–1823.

5. Tamura, K., Kurihara, H., Yonemori, K., Tsuda, H., Suzuki, J., Kono, Y., Honda, N., Kodaira, M., Yamamoto, H., Yunokawa, M., Shimizu, C., Hasegawa, K., Kanayama, Y., Nozaki, S., Kinoshita, T., Wada, Y., Tazawa, S., Takahashi, K., Watanabe, Y., Fujiwara, Y. *J. Nucl. Med.* **2013**, *54*, 1869–1875.
